# Biocompatible and Antimicrobial Cellulose Acetate-Collagen Films Containing MWCNTs Decorated with TiO_2_ Nanoparticles for Potential Biomedical Applications

**DOI:** 10.3390/nano12020239

**Published:** 2022-01-12

**Authors:** Madalina Elena David, Rodica Mariana Ion, Ramona Marina Grigorescu, Lorena Iancu, Alina Maria Holban, Florin Iordache, Adrian Ionut Nicoara, Elvira Alexandrescu, Raluca Somoghi, Sofia Teodorescu, Anca Irina Gheboianu

**Affiliations:** 1 National Institute for Research and Development in Chemistry and Petrochemistry—ICECHIM, 060021 Bucharest, Romania; rodica_ion2000@yahoo.co.uk (R.M.I.); rmgrigorescu@gmail.com (R.M.G.); lorenna77ro@yahoo.com (L.I.); elvira.alexandrescu@icechim-pd.ro (E.A.); ralucasomoghi@outlook.com (R.S.); 2 Doctoral School of Materials Engineering Department, Valahia University of Targoviste, 130004 Targoviste, Romania; 3 Faculty of Biology, University of Bucharest, 060101 Bucharest, Romania; alina_m_h@yahoo.com; 4 Department of Biochemistry, Faculty of Veterinary Medicine, University of Agronomic Science and Veterinary Medicine, 011464 Bucharest, Romania; floriniordache84@yahoo.com; 5 Faculty of Applied Chemistry and Materials Science, University Politehnica of Bucharest, 011061 Bucharest, Romania; adi.nicoara18@gmail.com; 6 Institute of Multidisciplinary Research for Science and Technology, Valahia University of Targoviste, 130004 Targoviste, Romania; sofiateodorescu@yahoo.com (S.T.); anca@icstm.ro (A.I.G.)

**Keywords:** wound dressing biomaterial, decorated carbon nanotubes, nanoparticles, antimicrobial properties

## Abstract

This research focuses on the synthesis of multi-walled carbon nanotubes (MWCNTs) decorated with TiO_2_ nanoparticles (NPs) and incorporated in cellulose acetate-collagen film in order to obtain a new biomaterial with potential biomedical applications and improved antimicrobial activity. The successful decoration of the MWCNTs with TiO_2_ NPs was confirmed by several structural and morphological analysis, such as Fourier transformed infrared spectroscopy, Raman spectroscopy, X-ray diffraction and transmission electron microscopy. The obtained nanocomposites were further incorporated into cellulose acetate-collagen films, at different concentrations and absorption kinetics, antimicrobial activity and in vitro biocompatibility of the obtained films was investigated. The antimicrobial tests sustained that the presence of the nanocomposites into the polymeric matrix is an important aspect in increasing and maintaining the antimicrobial activity of the polymeric wound dressings over time. The biocompatibility and cytotoxicity of the obtained films was evaluated using cellular viability/proliferation assay and fluorescent microscopy which revealed the ability of the obtained materials as potential wound dressing biomaterial.

## 1. Introduction

In the past several years, medicine has faced real challenges regarding the bacterial spread, mostly because bacteria have evolved in order to survive antimicrobial medications by developing resistance mechanisms [1]. This issue continues to remain a major problem for hospitals and contribute significantly to the rate of morbidity, mortality and cost of care, that aggravates the problem mainly in developing countries where resources are scarce and staffs are always in short supply [2,3]. Nosocomial infections are common in burn patients due to the typical features of the disease: loss of the first line of defence against microbial invasion; avascularised tissue that offers a promising environment for microbial development; modifications in the specific and nonspecific constituents of the immune system; and extended hospitalisation and therapeutic procedures [4,5]. Prolonged administration of antibiotics, often administrated in combination, results in selection of multidrug resistant nosocomial strains which belong mainly to several bacterial species, such as Gram-positive (i.e., *Staphylococcus aureus*, *Streptococcus sp.*, *Micrococcus sp.*) and Gram-negative (i.e., *Escherichia coli*, *Pseudomonas aeruginosa*) bacteria, and sometimes yeasts (i.e., *Candida albicans*) [6,7,8].

Classical treatment of infections involves systemic antibiotic administration for long periods of time, that lead to difficulties due to its low specificity, low efficiency and/or increased selection of bacterial resistance [8]. Worldwide, poor wound healing affects many people, due to the poorly regulated features of the healthy tissue repair response. In order to avoid these complications, new antibacterial systems based on polymers and nanoparticles were investigated. The ideal wound dressing should provide protection against bacterial infection, promote angiogenesis, provide a moist environment and enhance epidermal migration [9,10]. Natural polymers are generally accepted for wound-healing applications because of their ideal properties, but most of them highlight limited or no antimicrobial activity [11]. Polysaccharides are natural biopolymers obtained from plant sources, with excellent biocompatibility [12]. An example belonging to this class is cellulose acetate (CA) which has been used for the biomaterials expansion of various biomedical and tissue engineering applications [13]. CA provides excellent biocompatibility, biodegradability, but the most important characteristic is that it has the potential to improve the cellular interaction between fibroblast cells and biomaterial [13,14,15]. These characteristics have made CA the right candidate for wound dressing applications. In recent years, researchers have mixed CA with the other polymers in order to obtain a biomaterial with enhanced properties for wound dressing applications. Collagen is the most common fibril protein in the human constitution, of about 30% of the total protein mass. This protein serves both a structural role being the basic protein of connective tissues in skin and bone, and a functional role being involved in complex mechanisms of tissue growth and repair [11]. This protein attracts attention because of its good biocompatibility, facile biodegradation (its degradation products being absorbed without inflammation), permeability and establishing strong interactions between the cells (due to stimulation of specific cell-morphology phenotypes) [11,15].

Nanomaterials are immersed in polymeric wound coverage matrices with the purpose to offer antibacterial activity, enhanced the angiogenic potential and cell proliferation and thus, assuring fast wound healing [16,17]. Recently, carbon nanotubes (CNTs) and decorated carbon nanotubes have attracted great attention as additive in biopolymers for the development of novel composite biomaterials, due to their antimicrobial activity, relatively high biocompatibility, promote angiogenesis, unique chemical and physical properties [18,19,20]. Pristine CNTs may induce some toxic reactions, but it has been demonstrated that the addition of polar functional groups to the CNTs surface considerably reduces their toxicity. The CNTs are capable to damage the cell membrane in microorganisms by direct contact, which results in bacterial cell death. The bacteriostatic properties of CNTs are assigned due to their high surface/volume ratio and large inner volume [21]. Laganà P. investigated the antimicrobial properties of MWCNTs, both pristine and functionalised, at two concentrations (50 and 100 μg/mL^−1^), against bacterial strains isolated from hospital-acquired infections (*P. aeruginosa*, *K. Pneumoniae*, *E. coli* and *S. aureus*). It was reported that both types of MWCNTs and doses inhibited the bacterial strains and the functionalised MWCNTs exhibited a greater inhibiting effect, compared to pristine MWCNTs [22]. Moreover, it was demonstrated that the incorporation of a small concentration of MWCNTs (0.1%) in a polydimethylsiloxane (PDMS) matrix was able to reduce the *E. coli* adhesion with 20% [23]. When the concentration of MWCNTs was increased at 1% in the PDMS matrix a 60% reduction of *E. coli* adhesion was achieved [24]. In another study, it was demonstrated that Staphylococcus sp. did not grow on MWCNT composite films, suggesting that these films can inhibit microorganism attachment and biofilm formation on medical devices [25].

Presently, these nanotubes are being functionalised by different kinds of molecules or nanoparticles with the purpose of increasing drug delivery potential and improving their activity [26]. In the last years, MWCNTs have been successfully applied in the medical field, because CNTs exhibit better wound-healing properties comparing with other non-metallic nanomaterials, due to its property to promote cell migration when incorporated in hydrogels [27]. Ravanbakhsh H. and co-workers obtained an injectable hydrogel based on MWCNTs and glycol chitosan in order to investigate its potential on human dermal fibroblast cells (HDF). It was observed that small concentrations of CNT significantly increase cell migration in hydrogels, and accelerate tissue regeneration and wound healing in situations where there is insufficient migration in the unloaded matrix [20]. In another study, solubilised collagen Type I was used and polymerised in the presence of dispersed CNTs and HDF in order to obtain new biomaterials with HDF inserted directly in the matrix. After 7 days, it was reported that the viability of HDF was constantly increased, and the cell morphology was not perturbed by the existence of CNTs. Electrical conductivity of the constructs varied from 3 to 7 mS cm^−1^, depending on CNTs loading level, thus suggesting, that the electrical conductivity of cell-seeded collagen gels can be enhanced with the incorporation of CNTs [28]. Kittana N. and co-workers tested and compared the effect of chitosan complexed SWCNT and MWCNT hydrogels on 3T3 fibroblast cell line, in order to investigate the potential of these composites as wound-healing applications. The results sustained that the fibroblasts were viable in the presence of the complexes and were able to effectively organise and contract the extracellular matrix. Moreover, the composites were tested by in vivo on CC-72 line mice and it was observed that both types of complexes improved the re-epithelialisation of the wounds healing [26]. In another study, Murugesan B. and co-workers synthetised heteroatom (N, F, P/B)-incorporated MWCNTs by self-assembling ionic liquids in order to study their efficacy in wound healing. Their antibiofilm activity against *K. pneumoniae*, *P. aeruginosa*, *E. coli* and *B. subtilis* was investigated and the results revealed greater effectiveness for the obtained compositions, compared to pristine MWCNTs. Moreover, the synthesised materials were tested for its wound-healing ability in Wistar rats. It was reported that cells cultured on these materials displayed exceptional healing ability [29]. Das B. and co-workers studied the wound-healing potential of eco-friendly hyperbranched polyurethane and in situ prepared MWCNTs decorated with Fe_3_O_4_ nanoparticles. It was reported that the obtained dressing patch presented excellent in vivo wound-healing potency in albino mice with an enhanced wound-closure rate [30].

The aim of this study is to design and characterise hybrid nanomaterials based on MWCNTs_TiO_2_ incorporated in cellulose acetate-collagen film in order to obtain a potential wound dressings biomaterial with enhanced antimicrobial properties.

## 2. Materials and Methods

### 2.1. MWCNT_TiO_2_ Synthesis

MWCNTs used in this study were obtained by chemical synthesis, purified and functionalised in our previously study [31] and the decoration of the MWCNTs with nanoparticles was performed more easily. Due to the fact that the nanotubes surface is inert, a chemical treatment is required in order to make it more active to react with other chemical compounds. The obtained MWCNTs were used as template and stabiliser for nanoparticles formation in order to obtain the nanotubes decoration. The in situ decoration of MWCNTs was carry out by dispersing 0.03 g nanotubes into 30 mL isopropanol (C_3_H_8_O, *p* > 99%, CHIMREACTIV, Bucharest, Romania), under sonication for 1 h. Subsequently, 3 wt% titanium (IV) isopropoxide (C_12_H_28_O_4_TiA, *p* > 98%, ACROS Organics, Geel, Belgium) in 20 mL isopropanol was added dropwise into the MWCNTs solution, under vigorous stirring. The obtained mixture was stirred for 2 h, at room temperature and then the solution was filtered, washed and dried at 100 °C, for 2 h. Finally, the product was sintered at 500 °C, for 30 min.

### 2.2. Biocomposite Materials Synthesis

4% cellulose acetate (Fluka, Buchs, Switzerland) and 4% collagen from bovine (Sigma-Aldrich, Schnelldorf, Germany) were separately dissolved in 100 mL glacial acetic acid and water (70 mL and 30 mL, respectively) and stirred for 3 h. After the solutions have completely dissolved, the collagen solution was added into the cellulose acetate solution and stirred for 30 min. Then, 50 mL from the stock solution was cast in Petri dishes, in order to obtain cellulose acetate-collagen (CC) films. Moreover, 50 mL of the stock solution was mixed for 30 min with 0.01 g, 0.025 g and 0.05 g of MWCNT_TiO_2_, in order to obtain MWCNT_TiO_2_@CC films (according to Table 1). Samples were placed into Petri dishes, crosslinked with 1% glutaraldehyde (Merck, Darmstadt, Germany) by spraying and kept at room temperature for 48 h in order to evaporate the solvent.

### 2.3. Structural and Morphological Analyses

#### 2.3.1. Fourier Transformed Infrared Spectroscopy (ATR–FTIR)

ATR-FTIR was registered with a GX-type FTIR spectrometer (Perkin Elmer, Waltham, MA, USA), in the 4000–400 cm^−1^ range, 4 cm^−1^ resolution, by accumulating and mediating 32 spectra.

#### 2.3.2. Raman Spectroscopy Analysis

Raman spectroscopy was achieved with a Horiba equipment (Labram HR Evolution, Pailaiseau, France) having a 514 nm excitation wavelength and a 50× objective with a 10 s acquisition time.

#### 2.3.3. X-ray Diffraction Analysis (XRD)

A Rigaku Ultima IV diffractometer (Rigaku, Tokyo, Japan) using Cu K α radiation (λ = 1.54 Å), 40 kV accelerating voltage of the generator radiation and 30 mA emission current was used in order to obtain the XRD diffractograms. The diffractograms were recorded in parallel beam geometry over 2θ = 10° to 90° continuously at a scan rate of 4°/min.

#### 2.3.4. Transmission Electron Microscopy (TEM)

TEM analysis was performed by using a G2 F20 TWIN Cryo-TEM (Philips, Eindhoven, The Netherlands) with 200 keV accelerating voltage. Initially, the obtained samples were pre dispersed in distilled water and sonicated for one hour. For microscopy examination, one drop of the aqueous dispersion was placed on the holey formvar grid.

#### 2.3.5. Scanning Electron Microscopy (SEM)

The films morphology was investigated using an FEI Quanta Inspect FEG Scanning Electron Microscope (FEI, Hillsboro, OR, USA) with 30 kV accelerating voltage. The obtained films were previously sputtered with gold for 30 s.

### 2.4. Absorption Kinetics

Absorption kinetics of a film is an important parameter in tissue engineering, in order to confirm the stability of porous wound-dressings. This method offers evidence on how the film will act in contact with the body fluids and also, about their interaction that can limit the process of cellular differentiation [8]. In this study, the simulated body fluid (SBF) solution was obtained, according to the Kokubo’s methodology [32].

Each sample was cut into a cylinder of 3 cm diameter, weighed (initial mass, *W_i_*) and completely immersed in 20 mL SBF, at a temperature of 37° C for various periods of time. At specified time, the samples were taken off, the excess fluid from their surface was removed and weighed (mass at time *t*, *W_t_*). Absorption ratio was calculated according to Equation (1):

(1) $$Absorption\;ratio\;\%=\frac{W_{t}-W_{i}}{W_{i}}$$

### 2.5. Hardness Measurements

A Shore hardness durometer, model PosiTector SHD-A, DeFelsko, Ogdensburg, NY, USA was used in order to investigate the hardness of the obtained films. Ten measurements were recorded for each film and average value was calculated.

### 2.6. Antimicrobial Analysis

#### 2.6.1. Microbial Strains and Growth Conditions

*S. aureus* ATCC 25923, *E. coli* ATCC 25922 and *C. albicans* ATCC 10231, were obtained from American Type Culture Collection (ATCC, Manassas, VA, USA), and used in order to investigate the antimicrobial activity of the obtained biofilms. In order to obtain a fresh culture used for the subsequent studies, glycerol stocks were streaked on LB agar (for bacteria) or Sabouraud agar (for *C. albicans*). All experiments were implemented in triplicate.

#### 2.6.2. Qualitative Antimicrobial Assay—Growth Inhibition

An adapted diffusion test, with regard to the general rules exposed in the CLSI 2020 was used in order to qualitatively monitor the antibacterial activity of the obtained films.

Initially, a 0.5 McFarland bacterial suspension (1.5 × 10^8^ CFU/mL) was obtained in sterile saline (0.9% NaCl solution) and used as a standardised inoculum to swab inoculate Petri dishes containing nutritive agar. The films were cut (6 mm diameter) and sterilised by UV exposure for 30 min before use. The cut films were aseptically placed on the inoculated Petri dishes and incubated (37 °C for 20 h). After incubation, the diameter of growth inhibition developed around each film was measured and noted (in mm).

#### 2.6.3. Monospecific Biofilm Development

The antibiofilm performance was carried out by transferring the obtained films (6 mm in diameter, sterile) in sterile 24-well plates with 1 mL nutritive broth and inoculation of 10 μL of bacterial suspension of 0.5 McFarland standard density. Then, the as prepared plates were incubated (37 °C for 24 h). After that, the films were gently washed with 1 mL of sterile saline solution and then, the samples were transferred in 1.5 mL centrifuge tubes, in 1000 μL sterile saline solution, followed by vortexing the obtained films, for 30 s in order to ensure the detachment of biofilm cells in suspension. In order to evaluate the viable colony formation (CFU/mL), serial 10-fold dilutions were obtained and then inoculated on nutrient agar.

#### 2.6.4. Evaluation of the Planktonic Development of Microorganisms

Planktonic development in the company of the obtained films was studied in nutritive broth. Films of 6 mm in diameter were positioned in sterile 24-well plates and 1 mL of nutritive broth and 10 μL of the previously obtained 0.5 McFarland bacterial suspensions in PBS were added. Specimens were incubated at 37 °C for 24 h. The obtained bacterial culture (150 μL) was relocated to 96-well plates and the absorbance at 600 nm was spectrophotometrically investigated, in order to determine the development of planktonic (free-floating) cultures.

### 2.7. Statistical Analysis

Biological results were studied by the one-way ANOVA repeated measures test. The statistical analyses were achieved using GraphPad Prism Software, v. 9.2. The obtained results were compared by Tukey’s test (*p* < 0.05).

### 2.8. Cellular Viability Assays

#### 2.8.1. MTT Assay

MTT [3-(4,5-dimethylthiazolyl)-2,5-diphenyltetrazolium bromide] test (Vybrant^®^MTT Cell Proliferation Assay Kit, Thermo Fischer Scientific, Waltham, MA, USA) was used in order to investigate the biocompatibility of the obtained films. Human dermal fibroblast HDFn cells (ATCC, PCS-201-010, Manassas, VA, USA) were developed in DMEM medium (Sigma-Aldrich, Saint Luis, MO, USA) and supplemented with 10% foetal bovine serum, 1% antibiotics (penicillin and streptomycin) (Sigma-Aldrich, Saint Luis, MO, USA), changed twice a week. The HDFn cells were grown in 96-well plates, with a seeding density of 3000 cells/well in the presence of films, for 72 h. Then, 15 mL (12 mM) of MTT was added to the cells and incubated at 37 °C for 4 h. A solution of 1 mg sodium dodecyl sulphate in 10 mL HCl (0.01 M) was added and pipetted vigorously to solubilise the formed formazan crystals. A TECAN Infinite M200 spectrophotometer (Männedorf, Switzerland) was used to evaluate the optical density of the solubilised formazan, at 570 nm, after 1 h.

#### 2.8.2. Fluorescence Microscopy

In order to evaluate the biocompatibility of the obtained films a RED CMTPX fluorophore (Thermo Fischer Scientific, Waltham, MA, USA) was used. The CMTPX was added to the HDFn cell culture, in the presence of the obtained films. After 5 days, the viability and morphology of the HDFn were evaluated. The CMTPX fluorophore, at a concentration of 5 µM and incubated for 30 min, was added in the culture medium with the purpose to permit the dye penetration into the cells. Lastly, the HDFn cells were washed with PBS. An Olympus CKX 41 digital camera driven by CellSense Entry software (Olympus, Tokyo, Japan) was used to visualise the cells.

## 3. Results and Discussions

### 3.1. Characterisation of MWCNT_TiO_2_

The first stage of the research implies the characterisation of the MWCNT_TiO_2_ with the purpose of demonstrating the successful decoration of the nanotubes.

#### 3.1.1. FTIR Spectroscopy

**Figure 1:**
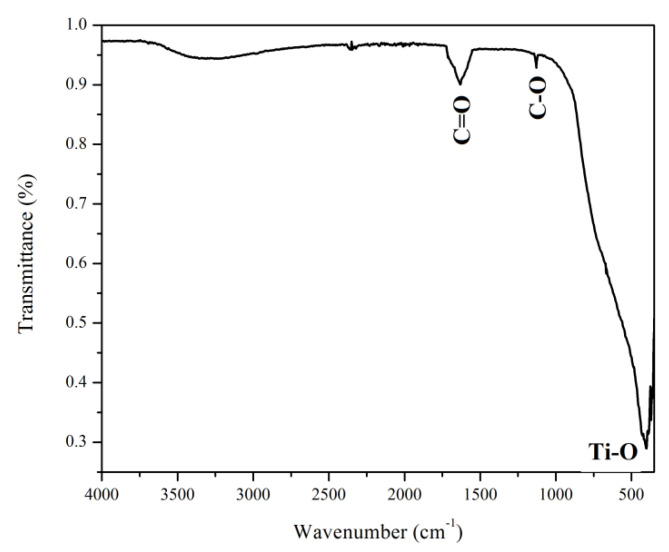
FTIR spectra of MWCNTs_TiO_2_.

FTIR spectra of MWCNTs_TiO_2_ confirms the presence of two important peaks, corresponding to C=C at 1632 cm^–1^ (carbon nanotubes’ skeleton) and Ti–O bonds at 419 cm^–1^, Figure 1. Moreover, the carbonyl group was confirmed by the peak at 1128 cm^–1^ corresponding to the C-O stretching. A broad absorption can be observed at approximately 3400 cm^−1^, probably due to water presence.

#### 3.1.2. Raman Analysis

**Figure 2:**
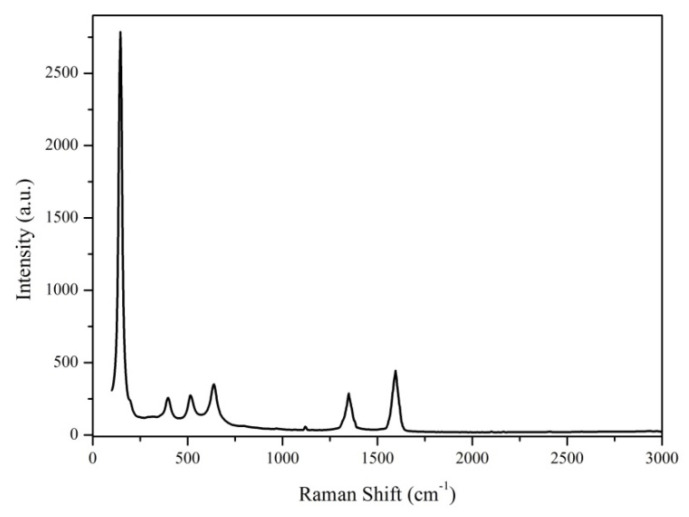
Raman spectra of MWCNTs_TiO_2_.

The Raman spectrum of MWCNT_TiO_2_ presented in Figure 2, confirms the specific band of anatase TiO_2_, such as 148.22 cm^–1^, 398.92 cm^–1^, 518.15 cm^–1^ and 639.31 cm^–1^. Moreover, the specific bands of MWCNTs are present, at 1597.63 cm^–1^ (G band), which refers to the crystalline nature of the MWCNTs and the band at 1346.29 cm^–1^ (D band), which indicates the distortions on the MWCNTs surface. The presence of D and G bands confirms the interfacial interaction between MWCNTs and TiO_2_ nanoparticles [33]. The ID/IG band ratio was calculated to be 0.842. Compared with the ID/IG band ratio of pure MWCNTs (0.839) [18], it can be suggested that the increasing of the ID/IG band ratio occurs because of the structural defects in the carbon wall, confirming thus, the modification of the outer layers of the MWCNTs by nanoparticles deposition.

#### 3.1.3. XRD Analysis

**Figure 3:**
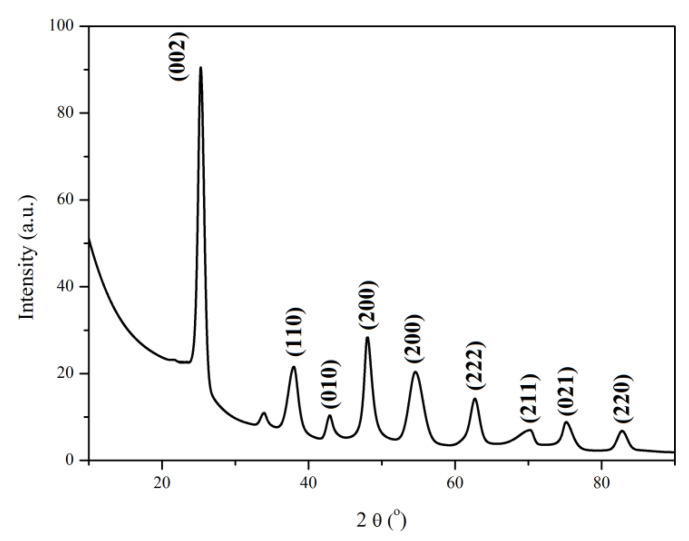
XRD pattern of MWCNTs_TiO_2_.

The XRD pattern of the MWCNTs_TiO_2_ (Figure 3) confirms the presence of the diffraction peaks corresponding to MWCNTs structure (at 25.26° and 42.88°). The characteristic diffraction peaks of TiO_2_ nanostructure observed at 37.99, 47.97, 54.54, 70.26, 75.04 and 82.71 are perfectly assigned to the crystal planes (110), (200), (211), (021) and (220) which confirm the anatase phase [34,35]. The peak at 25.26° corresponds to the crystal plane (002) reflection of MWCNTs and overlaps the crystal plane (101) reflection of anatase TiO_2_, peak that appears at 25.53°, confirming, thus, that the anatase phase was the major crystal in MWCNT/TiO_2_ nanocomposites.

Scherrer’s Equation (2) was used in order to calculate the crystallite size (L) of the MWCNTs_TiO_2_. Table 2 shows the crystallite size of the nanomaterials.

(2) L=Kλ/β cos θ

where, *K* is Scherrer constant (0.91), λ is the X-ray wavelength (1.5406 Å), *β* is the full-width at half maximum and *θ* is the Bragg angle (rad).

The significantly increased crystallite size in the case of MWCNTs_TiO_2_, compared to pure MWCNTs could be explained by the higher size of the TiO_2_ nanoparticles that covered the surface of the nanotubes (Table 2). MWCNTs_TiO_2_ shows the lowest distribution, that means largest crystallite size and lowest lattice strains [36]. The peak of decorated carbon nanotubes is four times narrower than MWCNTs indicating improved crystallinity that can impart higher mechanical properties in the nanocomposites.

#### 3.1.4. TEM Analysis

**Figure 4:**
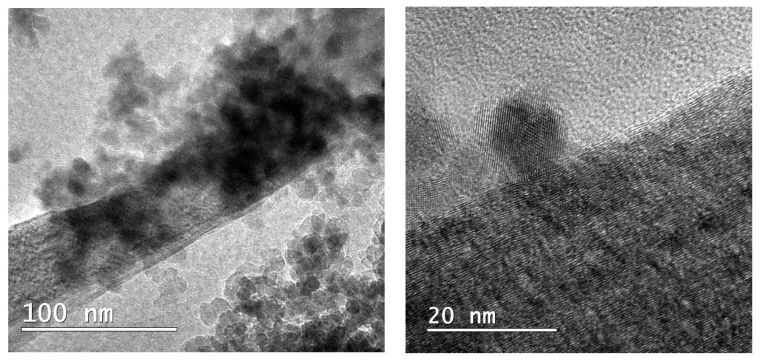
TEM micrographs of the obtained nanocomposites.

The nanotubes synthetised in our previous work [31] have a wide size distribution, with a diameter of 10–50 nm and approximately 600 nm in length, confirming thus, that the obtained MWCNTs were shorts. The synthetised nanotubes presented only a few defects on their surface, which suggests that nanotubes are composed of quality graphite layers. In Figure 4, the obtained MWCNTs_TiO_2_ are presented. The TiO_2_ NPs are successfully deposited on the surface of the nanotubes. The deposited nanoparticles have spherical shapes, with a diameter of about 15 nm. It can be observed that the nanoparticles are more intensely aggregated in some areas on the nanotubes surface.

### 3.2. Characterisation and Investigation of MWCNT_TiO_2_@CC Films

The second stage of the research implies the characterisation of the CC film and MWCNT_TiO_2_ incorporated in CC films (MWCNT_TiO_2_@CC) in order to investigate the potential of the films as wound dressing biomaterial. The obtained films presented a thickness of 0.148 mm (by calculating the arithmetic mean of 10 values).

#### 3.2.1. FTIR Spectroscopy

**Figure 5:**
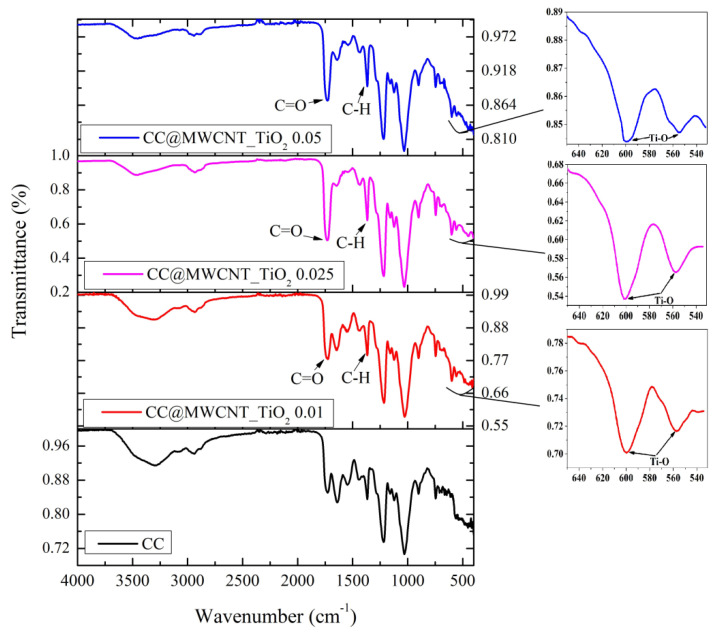
FTIR spectra of the obtained films.

The infrared spectrum of the obtained films is presented in Figure 5. The FTIR spectra of CC film demonstrate the existence of carbonyl group from the collagen, and the triple helix structure of collagen at 1638 cm^–1^—amide I, 1546 cm^–1^—amide II and at 1220 cm^–1^—amide III, from the N–H stretching of the hydrogen-bonded amide groups [13]. The presence of celluloses acetate is confirmed by its characteristic bands, such as the carbonyl vibration in the acetate substituent at 1730 cm^–1^, and the hydrogen oxygen vibration in hydroxyls or water presence at 3294 cm^–1^. The peak at 1359 corresponds to CH_3_ groups of the acetyl moiety and the peak at 1022 cm^−1^ matches to ether (C–O–C) bonds of the glycosidic bond [37].

By adding MWCNTs_TiO_2_, new characteristic peaks are observed, especially when the nanocomposite concentration increased. The FTIR spectra shows sharper peaks at 1371 cm^−1^ that are related to the C–H bonds characteristic of carbon nanotubes’ skeleton and 1730 cm^−1^ related to C=O from MWCNTs oxidation (indicating that carboxylic groups are formed due to the oxidation of some carbon atoms on the surface of the MWCNTs) [18,38]. The presence of TiO_2_ nanoparticles was confirmed by the band at low wavenumber around 605 and 558 cm^−1^.

#### 3.2.2. SEM Analysis

**Figure 6:**
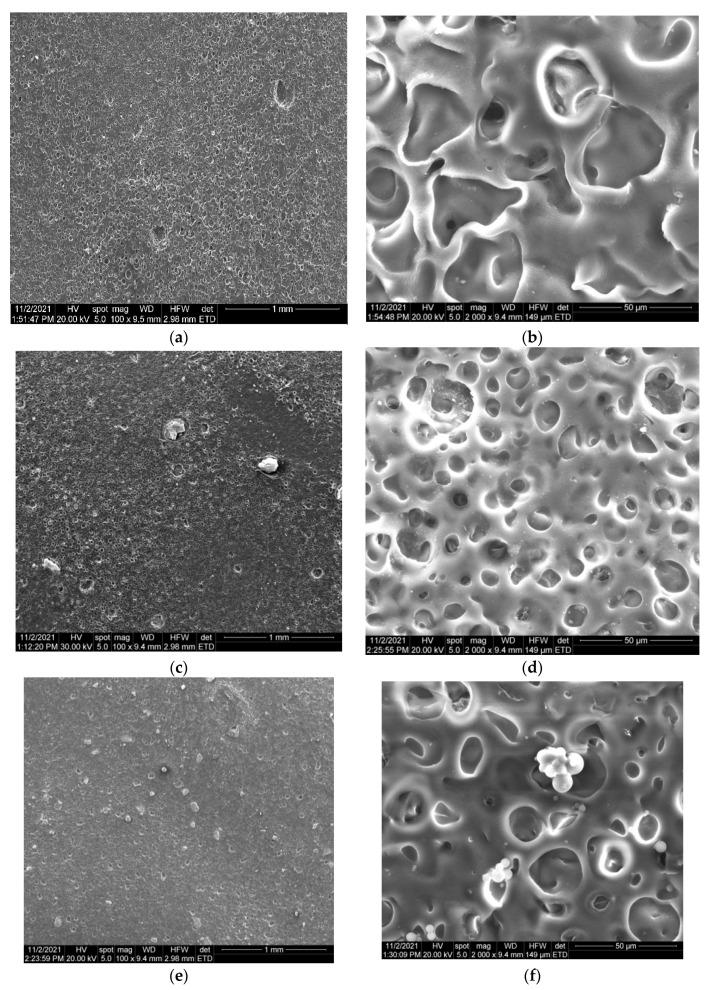

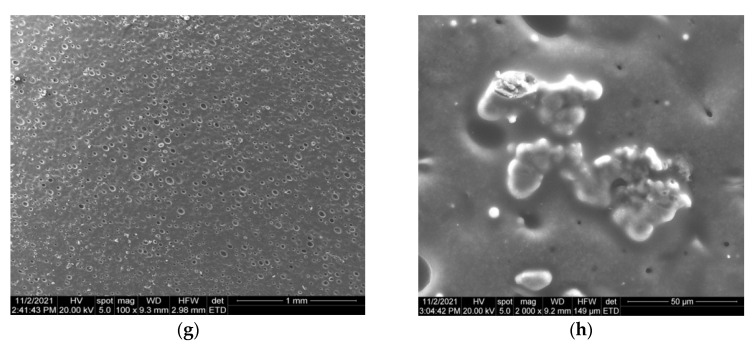
SEM micrographs showing the morphology of the films: CC (**a**,**b**), CC@MWCNT_TiO_2_ 0.01 (**c**,**d**), CC@MWCNT_TiO_2_ 0.025 (**e**,**f**) and CC@MWCNT_TiO_2_ 0.05 (**g**,**h**).

SEM micrographs of the obtained films are presented in Figure 6. Figure 6a,b illustrates the porous structure of the control sample (collagen and cellulose acetate without the antimicrobial agent). The porous film has mainly pore sizes of approximately 30 to 50 μm in diameter and smaller pores of approximately 5–10 μm. An open and interconnected relatively homogeneous porous structure can be observed for all the obtained films. When the antimicrobial agent was incorporated, a slight reduction of the pores dimensions was observed, mostly when the quantity of the agent increased. In all the cases, relatively homogeneous distribution of the agent can be observed. For the samples with increased concentration of nanocomposites, larger MWCNTs_TiO_2_ aggregates are presented (between 1.5–3 μm for CC@MWCNT_TiO_2_ 0.01 film; to 5–30 μm for CC@MWCNT_TiO_2_ 0.05 film).

#### 3.2.3. Absorption Kinetics

**Figure 7:**
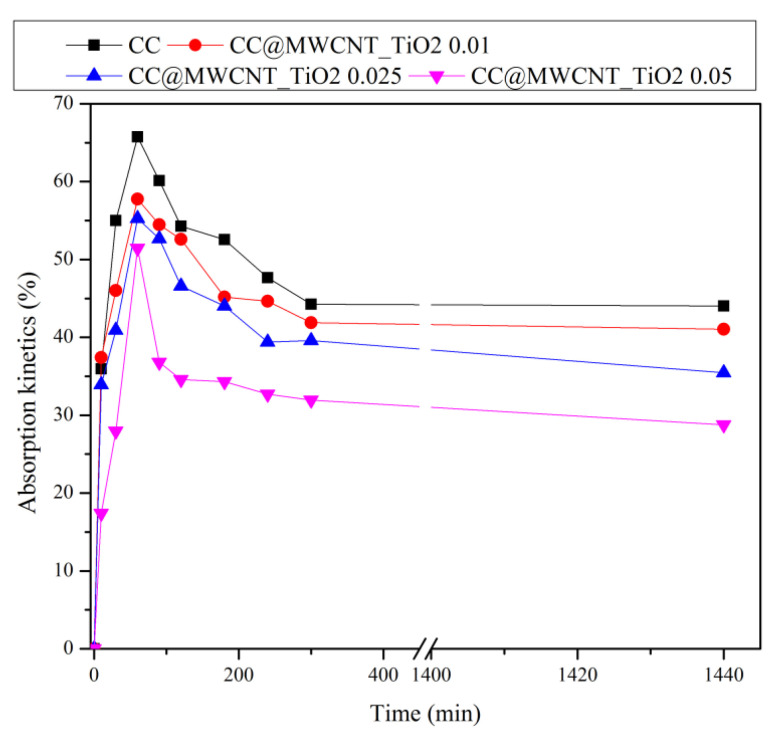
Absorption kinetics of the obtained films.

In Figure 7 are presented the absorption kinetics for the obtained films. It can be seen that the absorption kinetics of the films decrease with increase in the amount of MWCNTs_TiO_2_. This is attributed to the network structure formed between the nanomaterials and polymers, which prevent the absorption of a large quantity of water molecules. It can be observed that in the case of CC film, the maximum absorption of about 66% was reached after 60 min, while when the nanomaterials were incorporated lower absorption maxima can be observed. In the case of CC film, a slight decrease in absorption which stabilises after 300 min can be observed and also, this tendency is observed for the film with the lowest content of MWCNTs_TiO2 (0.01 g). When the nanocomposite content increased, lower maximum absorptions are observed, and absorption kinetics has a decreasing tendency over time.

#### 3.2.4. Hardness

**Figure 8:**
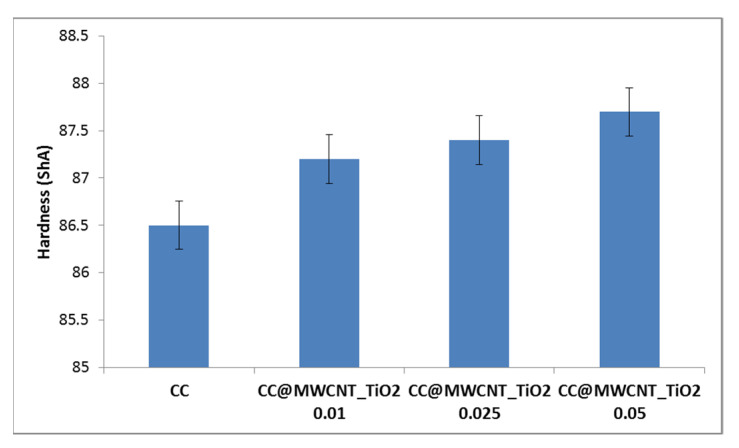
Hardness of the obtained films.

Maintaining a good mechanical character of the material is a very important feature of dressing. It has been reported in several studies that the incorporation of NPs into polymer composites significantly changes their mechanical properties [39]. Thus, the hardness of the obtained films was measured. As can be observed in Figure 8, the behaviour suggests that the bonding and microstructure of the films change significantly with increasing the MWCNTs_TiO_2_ content. It can be observed that even when a low content of MWCNTs_TiO_2_ was incorporated into the CC solution, effective hardness was obtained, compared to CC film. The large surface area of the nanomaterials leads to a stronger interfacial interaction between them and polymer molecules, and stress is better transferred through the materials which are subjected to mechanical effort, resulting in better properties of the CC films. Therefore, incorporating MWCNTs_TiO_2_ as reinforcing elements into the film could result in additional strength to the composite biomaterials.

#### 3.2.5. Antimicrobial Analysis

##### Qualitative Antimicrobial Assay—Growth Inhibition

**Figure 9:**
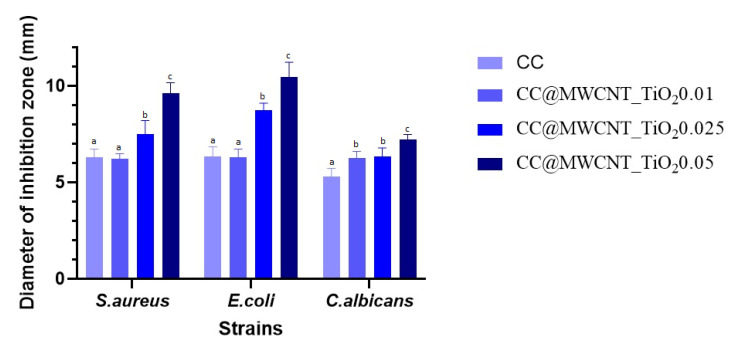
Graphic representation of growth inhibition diameters (mm) obtained after the cultivation of evaluated bacterial strains in the presence of films. Different small letters (a, b and c) indicate statistically significant differences between films (*p* < 0.05).

The obtained results demonstrated that the films presented a different antibacterial activity, depending both on the tested strain and the MWCNTs_TiO_2_ content (Figure 9). As can be observed, the highest values of growth inhibition zones were obtained for the film with the highest MWCNTs_ TiO_2_ content. The largest area of inhibition was obtained for *E. coli*, this can be related with the sensitivity of bacteria to nanocomposites that differs according to the interface provided by the bacterial membrane. It is well-known that Gram-positive bacteria are less sensitive to nanomaterials, compared to Gram-negative bacteria, because of their thicker peptidoglycan layer [40].

##### Monospecific Biofilm Development

**Figure 10:**
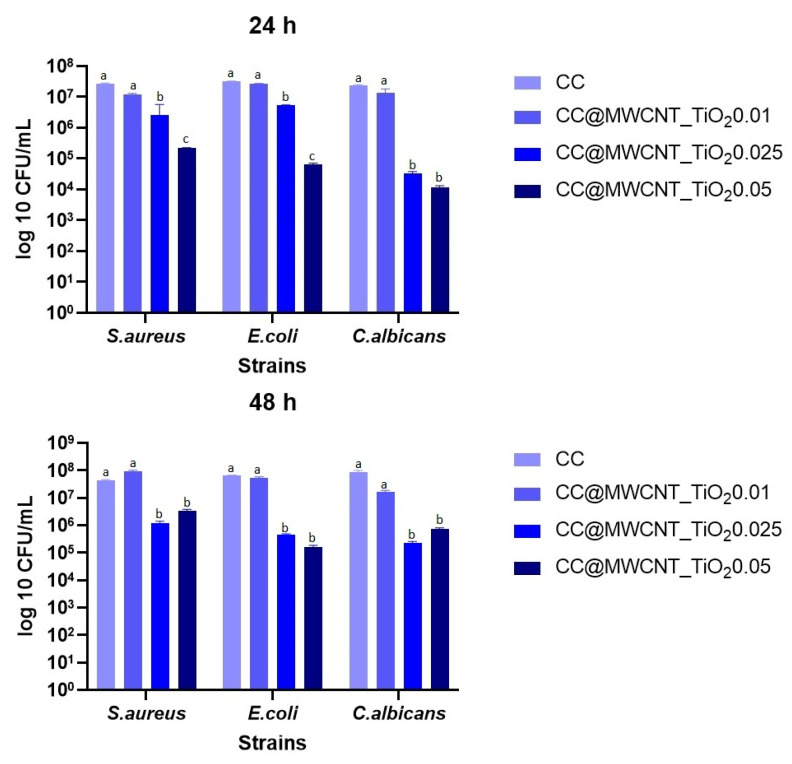
Values of log10 CFU/mL for the tested bacterial strains, expressing biofilm-embedded cells developed on the obtained films after 24 h (**up**) and 48 h (**bottom**) incubation. Different small letters (a, b and c) indicate statistically significant differences between films (*p* < 0.05).

The obtained results sustain that the biofilm expansion was significantly reduced at 24 h in all the cases of tested strains, when the films with the highest concentrations of nanocomposites were used (CC@MWCNTs_TiO_2_ 0.025 and CC@MWCNTs_TiO_2_ 0.05), so we can suggest that biofilm inhibition is dependent on the nanocomposites concentration (Figure 10). This tendency is maintained after 48 h, which suggests that the films are capable to offer protection over time. These results demonstrate that the presence of the hybrid nanomaterials is an important point. Many studies demonstrated the antimicrobial character of TiO_2_ NPs [41,42] and MWCNTs [18,43]. These data suggest that the films can combat specific pathogens, especially in wound-healing application, due to their higher effectiveness against Gram-positive and Gram-negative bacteria and yeast.

##### Evaluation of the Planktonic Development of Microorganisms

**Figure 11:**
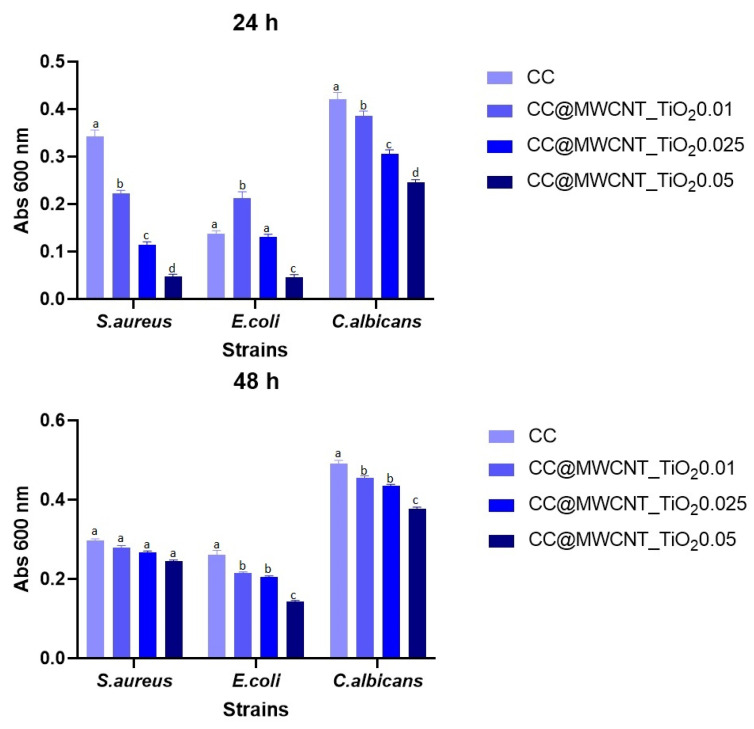
Absorbance at 600 nm indicating growth of planktonic cultures in the presence of films at 24 h (**up**) and 48 h (**bottom**) at 37 °C. Different small letters (a, b and c) indicate statistically significant differences between films (*p* < 0.05).

Planktonic development inhibition results showed growth inhibition in cases of *S. aureus* and *E. coli* when the nanocomposites are present, even at the lowest concentration. Figure 11 shows that the highest bacterial growth inhibition occurs for the film containing the highest amount of MWCNTs_TiO_2_. After 48 h, this tendency is maintained indicating that the antibacterial nanocomposites from the polymeric matrix could be released affecting the evolution of bacterial cells (Figure 11 bottom).

#### 3.2.6. Cellular Viability/Proliferation Assay (MTT Assay) and Fluorescence Microscopy

**Figure 12:**
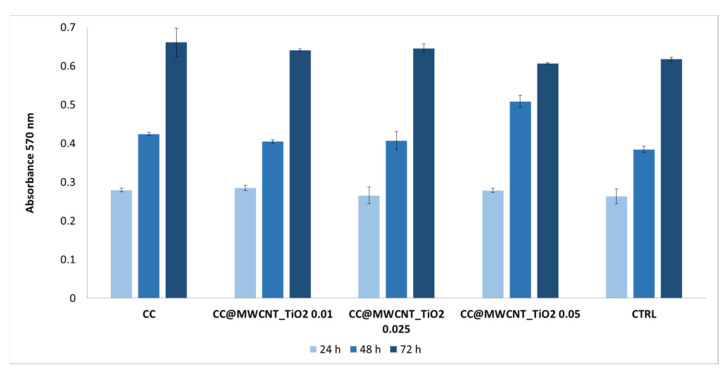
The viability of HDFn cells in the presence of films by MTT assay; the results are presented as the mean ±S.D. of three replicates.

MTT assay presented in Figure 12 shows that the obtained films present absorbance values close to the control sample at 24 h to 72 h, proving their non-toxicological effect. The metabolic activity of HDFn in the presence of films demonstrates to be higher than in the control cells, suggesting that cells are viable, improving thus the proliferation capacity. At 72 h, an increased proliferation capacity was observed for the films containing low and moderate quantity of nanocomposites (0.01 and 0.025 g MWCNTs_TiO_2_), compared to the control. These results sustain that the nanocomposites dose is one of the main factors in the relationships between the responding cell type and nanomaterials. An ideal biomaterial-based nanoparticles has to present the ability to minimise cytotoxicity to a range of potentially exposed cells. Regarding this, there are several reports that sustain that TiO_2_ nanoparticles and MWCNTs are non-toxic, and their exposure does not lead to membrane damage or cell death [44,45,46].

**Figure 13:**
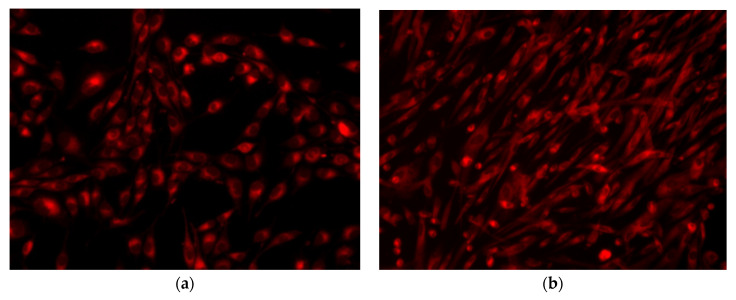

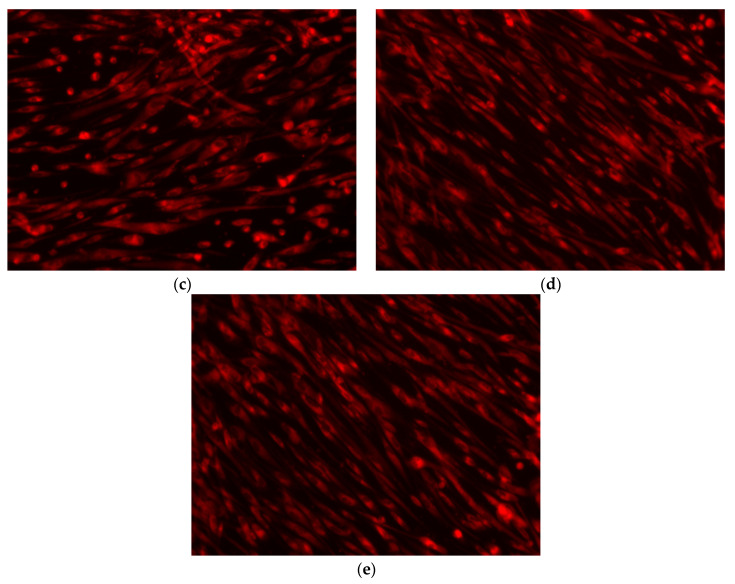
Fluorescence images showing the viability of HDFn cells coloured with CMTPX fluorophore: (**a**) control sample; (**b**) CC; (**c**) CC@MWCNT_TiO_2_ 0.01; (**d**) CC@MWCNT_TiO_2_ 0.025 and (**e**) CC@MWCNT_TiO_2_ 0.05 film.

Fluorescence microscopy images show that HDFn cells are viable, and the films have no cytotoxic effect, approving the biochemical assay. By fluorescence microscopy images it can be observed that the cellular metabolism of the cells is active, due to the fact that cells absorb the fluorophore CMTPX dye in the cytoplasm, demonstrating their viability after five days of incubation with HDFn cells. In Figure 13b no dead cells or cell fragments are detected when the cells are in contact with the film; the HDFn presents a normal morphology, with a normal fibroblast phenotype that preserves their initial morphology, with homogenous sizes and density distributions in the culture well plates. All the samples present extensions that demonstrate that the phenotype is active. In Figure 13d,e, HDFn is more abundant and has more elongated extensions suggesting that these materials stimulated the cells. This occurs because of the activity of the cytoskeleton and principally represents actin filaments and microtubules. As the HDFn cells are involved in several cellular processes, such as cell migration and wound healing, it can be concluded that the seeded cells demonstrated a good bioactivity for the studied films, especially when the concentration of MWCNTs_TiO_2_ increased.

## 4. Conclusions

In this study, we reported obtaining nanocomposites based on decorated MWCNTs with TiO_2_ NPs incorporated in cellulose acetate-collagen film in order to test their potential as antimicrobial wound dressing biomaterial. The decoration was confirmed by structural and morphological analyses, such as Fourier transformed infrared spectroscopy, Raman spectroscopy, X-ray diffraction and transmission electron microscopy. The attached nanoparticles on the nanotubes surface presented spherical shapes with diameter of about 15 nm. By TEM analysis it was observed that the nanoparticles are aggregated to the MWCNTs surface.

After obtaining the nanocomposites, they were incorporated in cellulose acetate-collagen film. The porous nature of the obtained biomaterials was established through SEM analysis, highlighting the micrometric pores, with homogenous distribution of TiO_2_ NPs into the polymeric matrix. The films absorbed a lower quantity of SBF when the MWCNTs_TiO_2_ quantity increased as demonstrated by absorption kinetics. This is attributed to the network structure formed between the nanomaterials and polymers, which prevents the absorption of large quantity of water molecules. The antimicrobial tests sustained that the presence of the nanocomposite in the polymeric matrix plays a key role in increasing and maintaining the antimicrobial activity of the polymeric wound dressings, over time. Moreover, the highest antimicrobial activity was obtained for the films with the highest concentrations of nanocomposites (CC@MWCNTs_TiO_2_ 0.025 and CC@MWCNTs_TiO_2_ 0.05). Overall, these films strongly inhibited the growth of both Gram-positive and Gram-negative strains, improving the cell proliferation capacity. Our results support the idea that the obtained films containing MWCNTs_TiO_2_ are biocompatible and possess antimicrobial activity, becoming thus successful candidates for the development of competent and efficient wound dressings. Further, in vivo studies will be designed to show the full range of bioactivity in our next paper.

## Figures and Tables

**Table 1:** Optimisation of films.

Sample No.	Sample Coding	Ratio of Cellulose Acetate/Collagen (g/g)	Observation
1	CC	1:1	-
2	CC@MWCNT_TiO_2_ 0.01	1:1	0.01 g of MWCNT_TiO_2_ was added over the solution
3	CC@MWCNT_TiO_2_ 0.025	1:1	0.025 g of MWCNT_TiO_2_ was added over the solution
4	CC@MWCNT_TiO_2_ 0.05	1:1	0.05 g of MWCNT_TiO_2_ was added over the solution

**Table 2:** Summary of the XRD characterisation of synthetised MWCNTs and MWCNTs_TiO_2_.

Sample	2θ (°)	2θ (rad)	β (°)	L (Å)	L (nm)
MWCNTs [18]	25.53	0.4456	4.08	20.18	2.018
MWCNTs_TiO_2_	25.267	0.2204	0.964	85.39	8.539

## Data Availability

Data is contained within the article.
